# The identification of high-performing antibodies for Sequestosome-1 for use in Western blot, immunoprecipitation and immunofluorescence

**DOI:** 10.12688/f1000research.132628.1

**Published:** 2023-03-23

**Authors:** Riham Ayoubi, Walaa Alshafie, Irina Shlaifer, Kathleen Southern, Peter S. McPherson, Carl Laflamme, NeuroSGC/YCharOS/EDDU collaborative group

**Affiliations:** 1 Department of Neurology and Neurosurgery, Structural Genomics Consortium, The Montreal Neurological Institute, McGill University, Montreal, Québec, H3A 2B4, Canada; 2 The Neuro’s Early Drug Discovery Unit (EDDU), Structural Genomics Consortium, McGill University, Montreal, Québec, H3A 2B4, Canada

**Keywords:** Uniprot ID Q13501, SQSTM1, Sequestosome-1, antibody characterization, antibody validation, Western blot, immunoprecipitation, immunofluorescence

## Abstract

Sequestosome-1, encoded by the gene *SQSTM1*, functions as a bridge between ubiquitinated proteins and the proteasome or autophagosome, thereby regulating protein degradation pathways. Loss of Sequestosome-1 is hypothesized to enhance neurodegeneration progression in several diseases, including amyotrophic lateral sclerosis (ALS) and frontotemporal disorders (FTD). Sequestosome-1 reproducible research would be facilitated with the availability of well-characterized anti-Sequestosome-1 antibodies. In this study, we characterized seventeen Sequestosome-1 commercial antibodies for Western blot, immunoprecipitation, and immunofluorescence using a standardized experimental protocol based on comparing read-outs in knockout cell lines and isogenic parental controls. We identified many high-performing antibodies and encourage readers to use this report as a guide to select the most appropriate antibody for their specific needs.

## Introduction

Sequestosome-1, alternatively known as p62, is an adaptor protein required for selective autophagy and proteasomal degradation. ^1^^–^^3^ Delivering polyubiquitinated proteins to the autophagosome or proteasome, Sequestosome-1/p62 plays a key role in the degradation of aggregate prone proteins. ^1^

*SQSTM1* gene mutations may act as a potential threat by causing altered autophagy, resulting in pathogenic protein aggregation and the development of a variety of neurodegenerative diseases, including ALS and FTD. ^4^ Furthermore, *SQSTM1* mutations have been identified in patients with ALS and FTD. ^5^ Serving as a signalling hub for neurodegenerative pathways, Sequestosome-1/p62 poses as a prospective therapeutic target in the treatment of neurodegenerative diseases. ^6^ Mechanistic studies would be greatly facilitated with the availability of high-quality antibodies.

Here, we compared the performance of a range of commercially available antibodies for Sequestosome-1 and validated high-performing antibodies for Western blot, immunoprecipitation and immunofluorescence, enabling biochemical and cellular assessment of Sequestosome-1 properties and function.

## Results and discussion

Our standard protocol involves comparing readouts from wild-type (WT) and knockout (KO) cells. ^7^^–^^9^ To identify a cell line that expresses adequate levels of Sequestosome-1 protein to provide sufficient signal to noise we examined public proteomics databases, namely PAXdb (RRID:SCR_018910) ^10^ and DepMap (RRID:SCR_017655). ^11^ U2OS was identified as a suitable cell line and thus U2OS was modified with CRISPR/Cas9 to knockout the corresponding *SQSTM1* gene ( Table 1).

**Table 1.** Summary of the cell lines used.

Institution	RRID (Cellosaurus)	Cell line	Genotype
Montreal Neurological Institute	CVCL_0042	U2OS	WT
Montreal Neurological Institute	CVCL_A6LP	U2OS	*SQSTM1* KO

**Figure 1.**
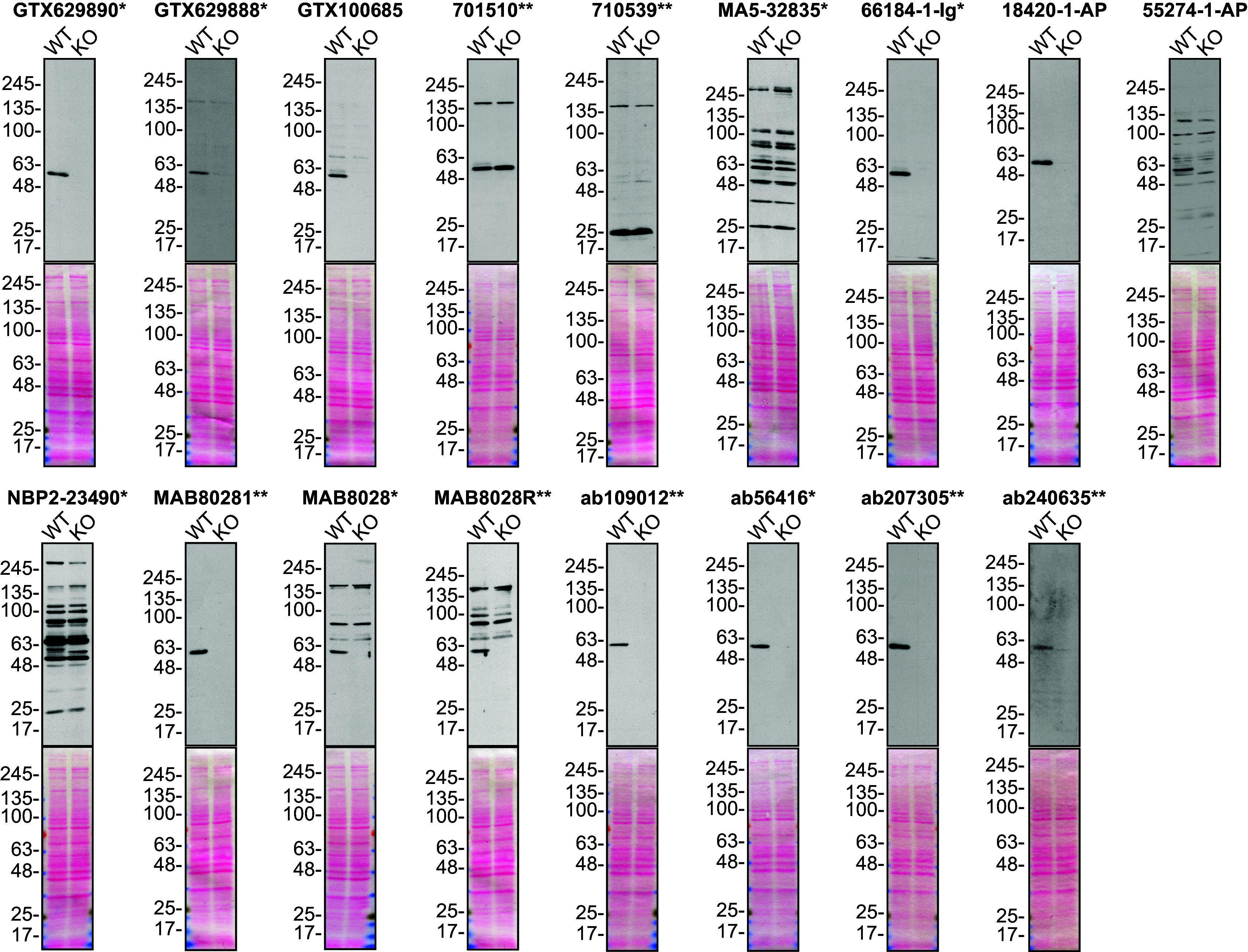
Sequestosome-1 antibody screening by Western blot. Lysates of U2OS (WT and *SQSTM1* KO) were prepared and 25 μg of protein were processed for Western blot with the indicated Sequestosome-1 antibodies. The Ponceau stained transfers of each blot are presented to show equal loading of WT and KO lysates and protein transfer efficiency from the acrylamide gels to the nitrocellulose membrane. Antibody dilutions were chosen according to the recommendations of the antibody supplier. Exceptions were given for antibodies MA5-32835*, 66184-1-Ig* and 18420-1-AP, which were titrated to 1/200, 1/1000 and 1/1000, respectively, as the signals were too weak when following the supplier’s recommendations. Antibody dilution used: GTX629890* at 1/1000; GTX629888* at 1/1000; GTX100685 at 1/1000; 701510** at 1/1000; 710539** at 1/200; MA5-32835* at 1/200; 66184-1-Ig* at 1/1000; 18420-1-AP at 1/1000; 55274-1-AP at 1/1000; NBP2-23490* at 1/1000; MAB80281** at 1/1000; MAB8028* at 1/1000; MAB8028R** at 1/1000; ab109012** at 1/10000; ab56416* at 1/1000; ab207305** at 1/1000; ab240635** at 1/1000. Predicted band size: ~62 kDa. *= monoclonal antibody, **= recombinant antibody.

For Western blot experiments, we resolved proteins from WT and *SQSTM1* KO cell extracts and probed them side-by-side with all antibodies in parallel ^8^^,^^9^ ( Figure 1).

**Figure 2.**
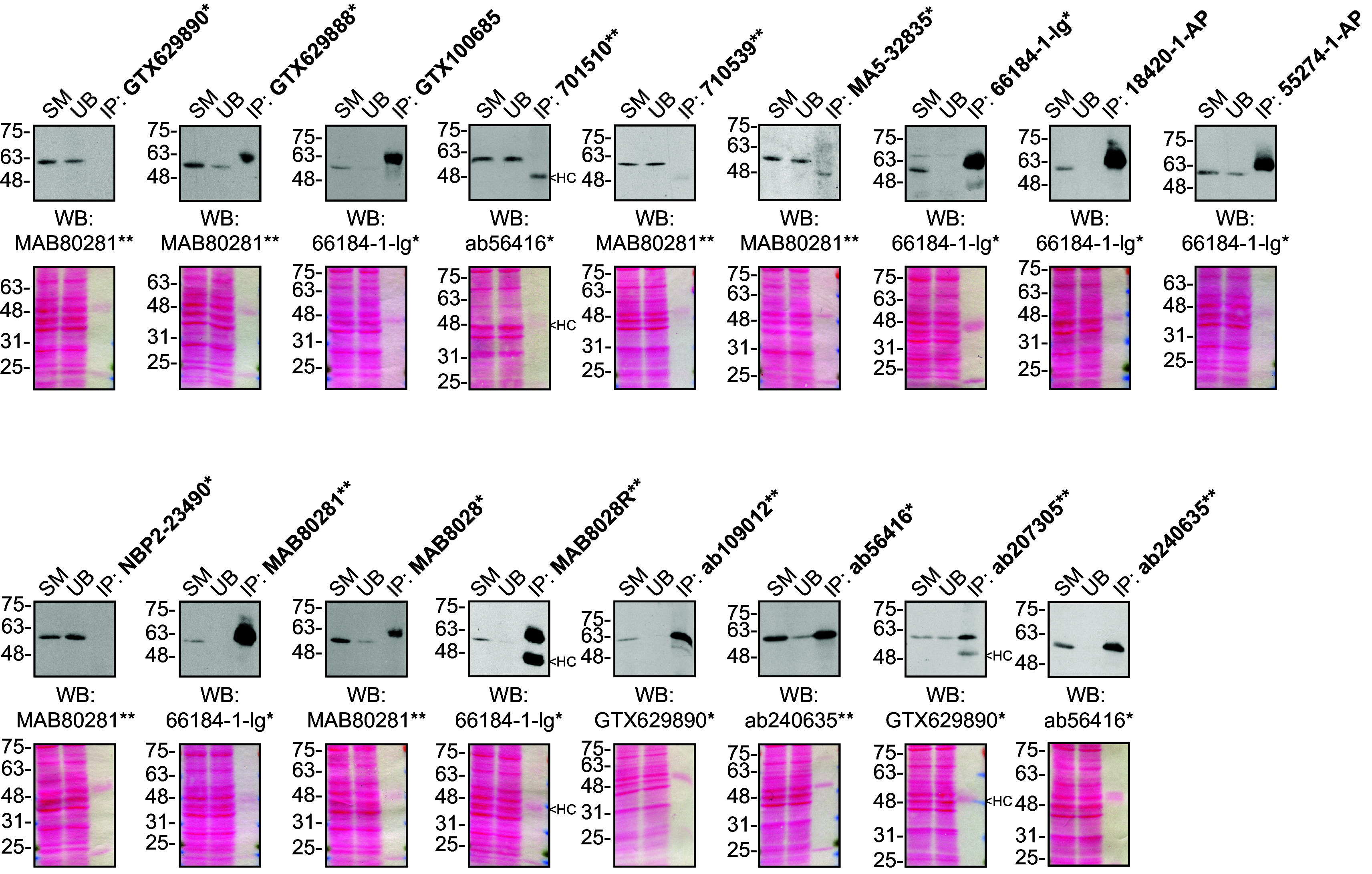
Sequestosome-1 antibody screening by immunoprecipitation. U2OS lysates were prepared, and IP was performed using 1.0 μg of the indicated Sequestosome-1 antibodies pre-coupled to protein G or protein A Sepharose beads. Samples were washed and processed for Western blot with the indicated Sequestosome-1 antibody. For Western blot, MAB80281** was used at 1/3000, 66184-1-lg* at 1/3000, ab56416* at 1/5000, ab207305** at 1/10000 and GTX629890* at 1/5000. The Ponceau stained transfers of each blot are shown for similar reasons as in Figure 1. SM= 10% starting material; UB=10% unbound fraction; IP=immunoprecipitate, HC= antibody heavy chain. *= monoclonal antibody, **= recombinant antibody.

For immunoprecipitation experiments, we used the antibodies to immunopurify Sequestosome-1 from U2OS cell extracts. The performance of each antibody was evaluated by detecting the Sequestosome-1 protein in extracts, in the immunodepleted extracts and in the immunoprecipitates ^8^^,^^9^ ( Figure 2).

**Figure 3.**
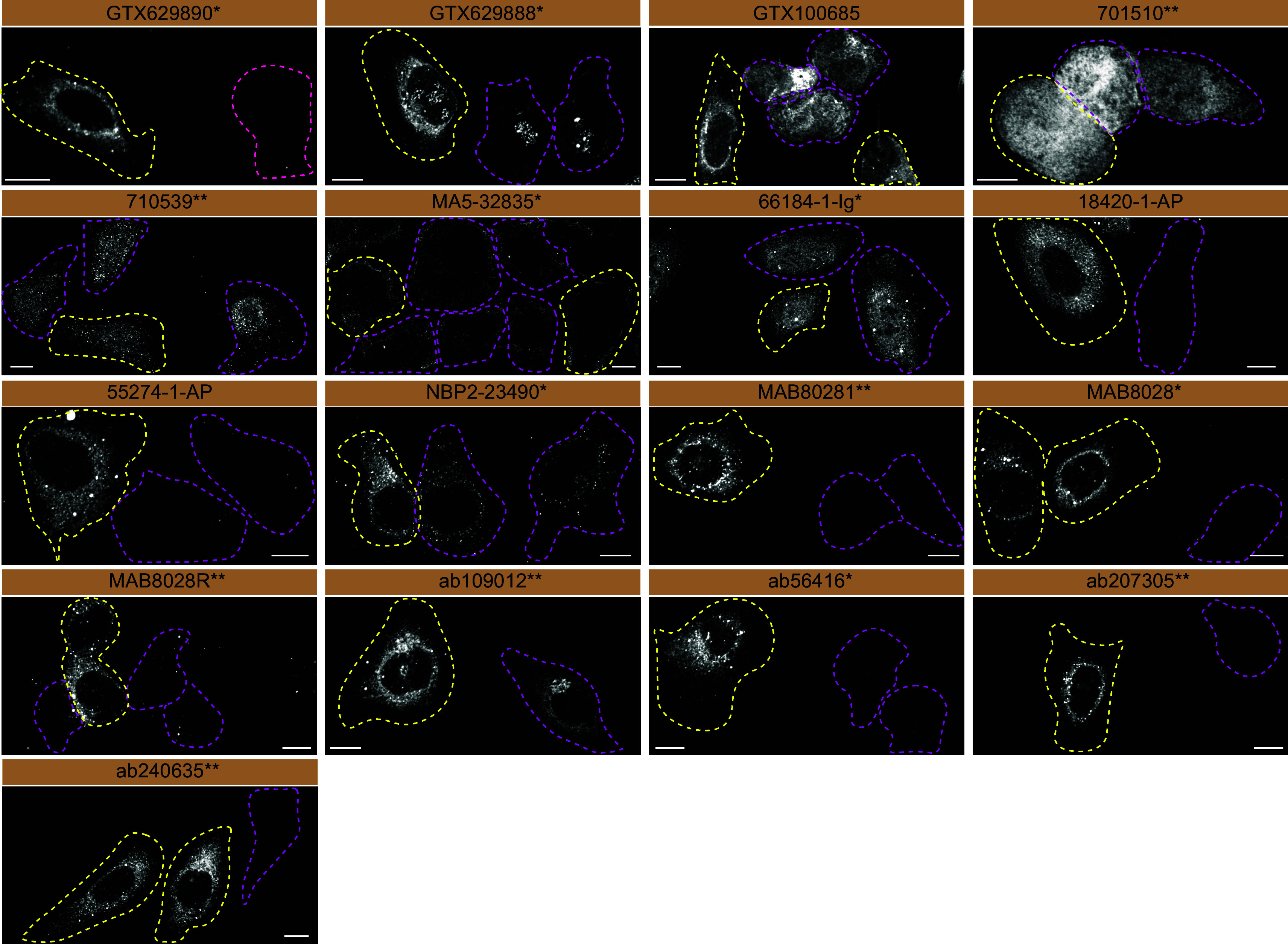
Sequestosome-1 antibody screening by immunofluorescence. U2OS WT and *SQSTM1* KO cells were labelled with a green or a far-red fluorescent dye, respectively. WT and KO cells were mixed and plated to a 1:1 ratio on coverslips. Cells were stained with the indicated Sequestosome-1 antibodies and with the corresponding Alexa-fluor 555 coupled secondary antibody. Acquisition of the green (WT), red (antibody staining) and far-red (KO) channels was performed. Representative images of red (grayscale images) channel are shown. WT and KO cells are outlined with yellow and magenta dashed line, respectively. Antibody dilutions were chosen according to the recommendations of the antibody supplier. Exceptions were given for antibodies MA5-32835*, 18420-1-AP and NBP2-23490*, which were titrated to 1/2000, 1/300 and 1/1000, respectively, as the signals were too strong when following the supplier’s recommendations. When the concentration was not indicated by the supplier, we tested antibodies at 1/500 and 1/1000. At this concentration, the signal from each antibody was in the range of detection of the microscope used. Antibody dilution used: GTX629890* at 1/1000; GTX629888* at 1/1000; GTX100685 at 1/700; 701510** at 1/500; 710539** at 1/500; MA5-32835* at 1/2000; 66184-1-Ig* at 1/300; 18420-1-AP at 1/300; 55274-1-AP at 1/1300; NBP2-23490* at 1/1000; MAB80281** at 1/500; MAB8028* at 1/500; MAB8028R** at 1/500; ab109012** at 1/500; ab56416* at 1/1000; ab207305** at 1/200; ab240635** at 1/500. Bars = 10 μm. *= monoclonal antibody, **= recombinant antibody.

For immunofluorescence, as described previously, antibodies were screened using a mosaic strategy. ^12^ In brief, we plated WT and KO cells together in the same well and imaged both cell types in the same field of view to reduce staining, imaging and image analysis bias ( Figure 3).

In conclusion, we have screened Sequestosome-1 commercial antibodies by Western blot, immunoprecipitation and immunofluorescence and characterized several high-quality antibodies under our standardized experimental conditions. The underlying data can be found on Zenodo. ^13^^,^^14^

## Methods

### Antibodies

All Sequestosome-1 antibodies are listed in Table 2, together with their corresponding Research Resource Identifiers, or RRID, to ensure the antibodies are cited properly. ^15^ Peroxidase-conjugated goat anti-rabbit and anti-mouse antibodies are from Thermo Fisher Scientific (cat. number 65-6120 and 62-6520). Alexa-555-conjugated goat anti-mouse and anti-rabbit secondary antibodies are from Thermo Fisher Scientific (cat. number A21424 and A21429).

**Table 2.** Summary of the Sequestosome-1 antibodies tested.

Company	Catalog number	Lot number	RRID (Antibody Registry)	Clonality	Clone ID	Host	Concentration (μg/μl)
GeneTex	GTX629890 *	41470	AB_2885144	monoclonal	GT1478	mouse	1.00
GeneTex	GTX629888 *	41470	AB_2885143	monoclonal	GT239	mouse	1.00
GeneTex	GTX100685	42893	AB_2038029	polyclonal	-	rabbit	0.67
Thermo Fisher Scientific	701510 **	2315239	AB_2532489	recombinant-mono	11HC14LC25	rabbit	0.50
Thermo Fisher Scientific	710539 **	RF229394	AB_2532735	recombinant-poly	11HCLC	rabbit	0.50
Thermo Fisher Scientific	MA5-32835 *	VL3152616	AB_2802482	monoclonal	10-E10	mouse	2.00
Proteintech	66184-1-Ig *	not provided	AB_2881579	monoclonal	1H5C1	mouse	1.33
Proteintech	18420-1-AP	not provided	AB_10694431	polyclonal	-	rabbit	0.35
Proteintech	55274-1-AP	not provided	AB_11182278	polyclonal	-	rabbit	0.43
Bio-Techne	NBP2-23490 *	A-1	AB_2885153	monoclonal	5H7E2	mouse	1.00
Bio-Techne	MAB80281 **	CMRM0120031	AB_2888658	recombinant-mono	2533b	rabbit	0.50
Bio-Techne	MAB8028 *	CHZL0520071	AB_2885150	monoclonal	864807	mouse	0.50
Bio-Techne	MAB8028R **	CLJP0118091	AB_2885151	recombinant-mono	864807R	mouse	0.50
Abcam	ab109012 **	GR3241806-8	AB_2810880	recombinant-mono	EPR4844	rabbit	0.43
Abcam	ab56416 *	GR3374761-2	AB_945626	monoclonal	Not provided	mouse	1.00
Abcam	ab207305 **	GR323335-4	AB_2885112	recombinant-mono	EPR18351	rabbit	0.21
Abcam	ab240635 **	GR3314160-2	AB_2885121	recombinant-mono	EPR23101-103	rabbit	0.49

Wb=Western blot; IF= immunofluorescence; IP=immunoprecipitation.

* = monoclonal antibody.

** = recombinant antibody.

### CRISPR/Cas9 genome editing

*SQSTM1* KO clone was generated in Cas9-expressing U2OS cell line ^7^ with low passage cells using an open-access protocol available on Zenodo.org: https://zenodo.org/record/3875777#.ZA9VdC-96Tf. Two guide RNAs were used to introduce a STOP codon in the *SQSTM1* gene (sequence guide 1: CCACCGCCCACCGUGUGCUC, sequence guide 2: AUGCGAGCUUGGUGUGCCCC).

### Cell culture

Both U2OS WT and *SQSTM1* KO cell lines used are listed in Table 1, together with their corresponding RRID, to ensure the cell lines are cited properly. ^16^ Cells were cultured in DMEM high glucose (GE Healthcare cat. number SH30081.01) containing 10% fetal bovine serum (Wisent, cat. number 080450), 2 mM L-glutamate (Wisent cat. number 609065), 100 IU penicillin and 100 μg/ml streptomycin (Wisent cat. number 450201).

### Antibody screening by Western blot

Western blots were performed as described in our standard operating procedure. ^17^ U2OS WT and *SQSTM1* KO were collected in RIPA buffer (50 mM Tris pH 8.0, 150 mM NaCl, 1.0 mM EDTA, 1% Triton X-100, 0.5% sodium deoxycholate, 0.1% SDS) supplemented with protease inhibitor (MilliporeSigma, cat. number P8340). Lysates were sonicated briefly and incubated for 30 min on ice. Lysates were spun at ~110,000 x g for 15 min at 4°C and equal protein aliquots of the supernatants were analyzed by SDS-PAGE and Western blot. BLUelf prestained protein ladder (GeneDireX, cat. number PM008-0500) was used.

Western blots were performed with large 4-15% polyacrylamide gels and transferred on nitrocellulose membranes. Proteins on the blots were visualized with Ponceau staining, which is scanned to show them together with individual Western blots. Blots were blocked with 5% milk for 1 hr, and antibodies were incubated overnight at 4°C with 5% bovine serum albumin (BSA) (Wisent, cat. number 800-095) in TBS with 0.1% Tween 20 (TBST) (Cell Signaling Technology, cat. number 9997). Following three washes with TBST, the peroxidase conjugated secondary antibody was incubated at a dilution of ~0.2 μg/ml in TBST with 5% milk for 1 hr at room temperature followed by three washes with TBST. Membranes were incubated with regular ECL (cat. number 32106) or super signal West Femto (cat. number 34096) from Thermo Fisher Scientific prior to detection with the HyBlot CL autoradiography films from Denville (cat. number 1159T41).

### Antibody screening by immunoprecipitation

Immunoprecipitation was performed as described in our standard operating procedure. ^18^ Antibody-bead conjugates were prepared by adding 1.0 μg of antibody to 500 μl of phosphate buffered saline (PBS) (Wisent, cat. number 311-010-CL) with 0.01% triton X-100 (Thermo Fisher Scientific, cat. number BP151-500) in a 1.5 mL microcentrifuge tube, together with 30 μl of protein A- (for rabbit antibodies) or protein G- (for mouse antibodies) Sepharose beads. Tubes were rocked overnight at 4°C followed by several washes to remove unbound antibodies.

U2OS WT were collected in HEPES lysis buffer (20 mM HEPES, 100 mM sodium chloride, 1 mM EDTA, 1% Triton X-100, pH 7.4) supplemented with protease inhibitor (MilliporeSigma, cat. number P8340). Lysates were rocked 30 min at 4°C and spun at 110,000 x g for 15 min at 4°C. One ml aliquots at 1.0 mg/ml of lysate were incubated with an antibody-bead conjugate for ~2 hours at 4°C. The unbound fractions were collected, and beads were subsequently washed three times with 1.0 ml of HEPES lysis buffer and processed for SDS-PAGE and Western blot on a 4-15% polyacrylamide gels as described above.

### Antibody screening by immunofluorescence

Immunofluorescence was performed as described in our standard operating procedure. ^8^^,^^9^^,^^12^ U2OS WT and *SQSTM1* KO were labelled with a green and a deep red fluorescence dye from Abcam (cat. number ab176735 and ab176736), respectively. WT and KO cells were plated on glass coverslips as a mosaic and incubated for 24 hrs in a cell culture incubator at 37°C, 5% CO _2_. Cells were fixed in 4% paraformaldehyde (PFA) (Beantown chemical, cat. number 140770-10ml) in PBS for 15 min at room temperature and then washed three times with PBS. Cells were permeabilized in PBS with 0.1% triton X-100 for 10 min at room temperature and blocked with PBS with 5% BSA, 5% goat serum (Gibco, cat. number 16210-064) and 0.01% Triton X-100 for 30 min at room temperature. Cells were incubated with IF buffer (PBS, 5% BSA, 0.01% Triton X-100) containing the primary Sequestosome-1 antibodies overnight at 4°C. Cells were then washed 3 x 10 min with IF buffer and incubated with corresponding Alexa Fluor 555-conjugated secondary antibodies in IF buffer at a dilution of 1.0 μg/ml for 1 hr at room temperature. Cells were washed 3 x 10 min with IF buffer and once with PBS. Coverslips were mounted on a microscopic slide using fluorescence mounting media (DAKO).

Imaging was performed using a Zeiss LSM 880 laser scanning confocal microscope equipped with a Plan-Apo 40x oil objective (NA = 1.40). Analysis was done using ImageJ (RRID:SCR_003070). All cell images represent a single focal plane. Figures were assembled with Adobe Photoshop (version 24.2.1) (RRID:SCR_014199) to adjust contrast and apply 1-pixel Gaussian blur, and then they were assembled with Adobe Illustrator (version 27.3.1) (RRID:SCR_010279).

## Data Availability

Zenodo: Antibody Characterization Report for Sequestosome-1, https://doi.org/10.5281/zenodo.4731001. ^13^ Zenodo: Dataset for the Sequestosome-1 antibody screening study, https://doi.org/10.5281/zenodo.7709902. ^14^ Data are available under the terms of the Creative Commons Attribution 4.0 International license (CC-BY 4.0).
