# Supplementary material for: The identification of high-performing antibodies for Sequestosome-1 for use in Western blot, immunoprecipitation and immunofluorescence
Source: F1000Res. 2024 Jul 10;12:324. Originally published 2023 Mar 23. [Version 2] doi: 10.12688/f1000research.132628.2 (PMC11240082; doi:10.12688/f1000research.132628.2)
Supplement: Peer review history

# Reviewer report: Reviewer response for version 2

**DOI:** 10.5256/f1000research.168891.r301249

**Published:** 2024-07-11

**Authors:** Guoqiang Xu

**Affiliations:** 1 soochow university, Jiangsu, China

This is a useful resource although it is not perfect.

Are sufficient details of methods and materials provided to allow replication by others?

Yes

Is the rationale for creating the dataset(s) clearly described?

Yes

Are the datasets clearly presented in a useable and accessible format?

Yes

Are the protocols appropriate and is the work technically sound?

Yes

Reviewer Expertise:

biochemistry, pharmacology, cancer biology, proteomics

I confirm that I have read this submission and believe that I have an appropriate level of expertise to confirm that it is of an acceptable scientific standard.

# Reviewer report: Reviewer response for version 1

**DOI:** 10.5256/f1000research.145562.r247595

**Published:** 2024-02-21

**Authors:** Guoqiang Xu

**Affiliations:** 1 soochow university, Jiangsu, China

This paper used the WT and Sequestosome-1 knockout U2OS cells to evaluate the quality of the anti-Sequestosome-1 antibodies obtained from different companies in three different applications, i.e. WB, IP, and IF.  The results are very useful for other scientists to choose the right antibody for the specific application.

 In the paper, WT and Sequestosome-1 knockout U2OS cells were used for the experiment. Additional cell lines, especially for different cancer cell lines and neuronal cell lines should also be tested since the antibodies will be most probably used in these cell lines in real situations.  In addition, different cell lines have different levels of protein expression and post-translational modifications, which are also frequently affecting the experimental results.

 For Figure 2, why do only two blots have bands for Heavy chains (HC)?  An explanation should be provided in the figure legend or the text for better understanding.

 For Figure 3, it is recommended that the authors provide the green, red, and contrast images for each antibody for better visualization and assessment.

 A conclusion should be provided at the end of the paper to show the quality and application of the different antibodies tested in this paper.  A comparison among different antibodies is very valuable for other scientists.  In addition, it will be useful to discuss the possible advantages and disadvantages of this work.

Are sufficient details of methods and materials provided to allow replication by others?

Yes

Is the rationale for creating the dataset(s) clearly described?

Yes

Are the datasets clearly presented in a useable and accessible format?

Yes

Are the protocols appropriate and is the work technically sound?

Yes

Reviewer Expertise:

biochemistry, pharmacology, cancer biology, proteomics

I confirm that I have read this submission and believe that I have an appropriate level of expertise to confirm that it is of an acceptable scientific standard, however I have significant reservations, as outlined above.

# Response

**Published:** 2024-07-05

**Authors:** Kathleen Southern

**Affiliations:** McGill University, Canada

Thank you Guoqiang Xu for taking the time to thoroughly review this Data Note, we appreciate your constructive feedback and hope our responses relieve any concerns or questions you previously had. As such, a new version of this article has been submitted, to ensure transparency and prevent further misinterpretations. We trust that the refinements made meet your expectations, enhance comprehensibility and address any concerns you might have had. Please see our responses to your specific comments below. 

**In the paper, WT and Sequestosome-1 knockout U2OS cells were used for the experiment. Additional cell lines, especially for different cancer cell lines and neuronal cell lines should also be tested since the antibodies will be most probably used in these cell lines in real situations.  In addition, different cell lines have different levels of protein expression and post-translational modifications, which are also frequently affecting the experimental results.**

 Response: We understand how the cell line selection process was not well understood, and we have edited the first paragraph of the Results and discussion section to clarify this part of our protocol. From analyzing the public expression database on Depmap, we found that HAP1 expressed PPP2R5D transcript at 6.7 log _2_ (TPM+1), which is significantly above the minimum threshold level, 2.5 log _2_ (TPM+1), previously established. A limitation of the orthogonal knockout (KO) based approach to test antibodies is that only one cell line (WT and KO) is used to evaluate the performance of the antibodies.  However, the strength of our approach lies in the simultaneous testing of multiple antibodies from a variety of commercial sources using WT and an equivalent KO cell line, aimed at identifying the most suitable antibodies for specific applications. A detailed description of the orthogonal strategy applied to select cell lines for the study is outlined in our antibody characterization platform, available on Protocol exchange and (DOI: 10.21203/rs.3.pex-2607/v1). This reference has been included in the new version.

**For Figure 2, why do only two blots have bands for Heavy chains (HC)?  An explanation should be provided in the figure legend or the text for better understanding.**

 Response: In the protocol exchange paper, previously referred to, which outlines the antibody characterization procedure followed in this article, and is now referenced in the new version of the article. Within the protocol, we have a troubleshooting section with proposed solutions to prevent the heavy chains from appearing. It can be found in the IP Procedure section 2E). Although we have tried to optimize the workflow, we cannot control whether the heavy chain bands appear and show the IP results as we do with all the other antibodies tested.

**For Figure 3, it is recommended that the authors provide the green, red, and contrast images for each antibody for better visualization and assessment.**

 Response: This underlying data is provided in the Dataset, found in the **Data availability **section. You can also use the following link to be redirected to the Dataset, https://doi.org/10.5281/zenodo.7709902.

**A conclusion should be provided at the end of the paper to show the quality and application of the different antibodies tested in this paper.  A comparison among different antibodies is very valuable for other scientists.  In addition, it will be useful to discuss the possible advantages and disadvantages of this work.**

 Response: The authors do not participate in results analysis nor offer explicit antibody recommendations, therefore preventing them from identifying which antibodies were high-performing in each respective application. Due to the confines of the experimental setup, the factors that can influence the performance of antibodies and cell line used, we do not draw conclusions. We leave this analysis up to the viewers and within our gateway we feature an editorial that can be used as a guide on how the scientific community can utilize and analyze the YCharOS data to identify high-performing antibodies within the confines of our protocol (DOI: 10.12688/f1000research.141719.1).

 Furthermore, given that that the article is formatted as a Data Note, it does not require the results to be discussed or concluded.

 That being said, we understand how this aspect of the YCharOS initiative was not clearly defined and in the newly submitted version we have included two paragraphs to the end of the introduction that will provide an understanding as to why we do not analyze the results, preventing further misinterpretation.

# Reviewer report: Reviewer response for version 1

**DOI:** 10.5256/f1000research.145562.r247622

**Published:** 2024-02-21

**Authors:** Yasukazu Takanezawa

**Affiliations:** 1 Kitasato University, Tokyo, Japan

This paper delves into the intricacies of Western blot, immunoprecipitation, and immunofluorescence techniques utilizing commercially available antibodies targeting p62, particularly in the context of neurodegenerative disease pathogenesis. This paper provides valuable insights into the application of p62 antibodies. Additionally, incorporating co-staining with LC3 would enhance its informativeness. Moreover, exploring the characterization of anti-p62 antibodies from other companies would be advantageous.

Are sufficient details of methods and materials provided to allow replication by others?

Yes

Is the rationale for creating the dataset(s) clearly described?

Yes

Are the datasets clearly presented in a useable and accessible format?

Yes

Are the protocols appropriate and is the work technically sound?

Yes

Reviewer Expertise:

As a specialist in toxicology, particularly focused on the interplay between autophagy and heavy metals.

I confirm that I have read this submission and believe that I have an appropriate level of expertise to confirm that it is of an acceptable scientific standard.
